# Neoplasia and Proliferative Lesions of New World Camelids: A Systematic Literature Review and Retrospective Study of Cases Submitted to Colorado State University From 1995 to 2020

**DOI:** 10.3389/fvets.2021.743498

**Published:** 2021-10-22

**Authors:** Tawfik A. Aboellail, Max Waugh, Alexandra Harvey, Jade Fisher, Allison C. Vilander

**Affiliations:** ^1^Department of Microbiology, Immunology and Pathology, College of Veterinary Medicine and Biomedical Sciences, Colorado State University, Fort Collins, CO, United States; ^2^Department of Pathobiology, School of Veterinary Medicine, University of Pennsylvania, Philadelphia, PA, United States; ^3^Antech Diagnostics, Fountain Valley, CA, United States

**Keywords:** new world camelid, alpaca, llama, neoplasia, proliferative lesions

## Abstract

Camelid pathology submissions to veterinary diagnostic laboratories are on the rise given the increasing popularity and population of llamas and alpacas especially in the western United States. When compared to other animals, the field of camelid neoplasia has a relative paucity of cases reported in the literature. The Colorado State University Veterinary Diagnostic Laboratories (CSU-VDL) has had a steady increase in the numbers of camelid pathology submissions allowing for a robust review of diagnoses of neoplasia in new world camelids. Here we present a retrospective analysis of camelid neoplastic and proliferative lesions diagnosed at the CSU-VDL from 1995 to 2020, followed by an extensive literature review. Results show increasing incidence of camelid neoplasia reported in the literature, therefore becoming a common diagnosis in llamas and alpacas. Proliferative and neoplastic lesions were diagnosed in 8.8% of new world camelid submissions to CSU-VDL with the most common tumors being lymphomas, squamous cell carcinomas, fibromas, and adenocarcinomas. Risk factors are female sex and increased age except in the case of lymphoma, which tends to occur in younger camelids. Lymphomas, melanomas, and adenocarcinomas (especially of gastrointestinal tract) carry an increased risk of multiple-organ system involvement often with widespread metastases. Conditions described in camelids for the first time include osteosarcoma, cutaneous hemangiosarcoma, myxosarcoma, pilomatricoma, ovarian theca cell tumor, congenital nevus with malignant transformation, and various other neoplasia. This article will provide an operational guide for camelid neoplasia to further assist veterinary laboratory diagnosticians, researchers, and practicing veterinarians in the field of camelid medicine and pathology.

## Introduction

The first report of neoplasia in new world camelids, published in 1974, described gastric squamous cell carcinoma in a guanaco (1). Since then, there has been increasing interest in neoplasia of llamas and alpacas especially as these animals have had increased popularity for their fiber and as companion and pack animals for integrated sheep protection. The majority of the earlier reports have been individual cases or brief communications with only very rare publications looking at overall prevalence of neoplasia in new world camelids (2, 3). Prevalence studies indicate that while common, neoplasia is not a primary cause of death and that llamas might have a slightly higher incidence of neoplasia than alpacas, although alpacas appear to have predisposition for developing neoplasia at a relatively younger age. Publications of neoplasia in camelids often report disseminated or metastatic disease at the time of diagnosis, making early diagnosis especially important in these animals.

Sample size in most of the reports in new world camelid literature is limited with only 6% of publications (5 of 83 publications) on neoplasia in camelids reporting numbers greater than five animals. Case reports are rather common and often report bizarre or highly aggressive neoplastic behavior discovered at the time of necropsy, leaving a gap in the current knowledge as to the outcome of these tumors. Pathologists, clinicians, and owners are often challenged with prognostication relating to cutaneous or visceral neoplasia that have been underreported or previously unreported. A guide to differentiate benign from malignant tumors is of crucial importance in those cases. A systematic review of neoplasia, to the authors' knowledge, has yet to be published on neoplasia in llamas and alpacas.

The current article presents a systematic review of neoplasia and proliferative diseases in new world camelids including cases from both the published literature and 201 case submissions to the Veterinary Diagnostic Laboratory at Colorado State University (CSU-VDL) from 1995 to 2020. The case series published here is the largest summary data ever reported on this subject and almost doubles the number of the known cases of neoplasia reported in South American camelids. This review will serve to broaden the knowledge base on camelid neoplasia and will highlight the most common neoplastic diseases and likelihood of malignancy and will also identify novel neoplastic/proliferative lesions in new world camelids.

## Materials and Methods

### Identification of Case Submissions

Query of camelid (alpaca, llama, or unspecified camelid) necropsy and biopsy cases submitted to the CSU-VDL from 1995 to 2020 identified 2,166 submissions. Review of these submissions identified 201 diagnoses (from 192 animals) of neoplasia or proliferative lesions. Results are summarized in Table 1. For retrospective evaluation of the types and frequencies of different camelid neoplasia, biopsy, and necropsy reports were reviewed, and in cases of previously undescribed proliferative or neoplastic disease the hematoxylin and eosin-stained (H&E) slides archived at CSU-VDL were pulled and reviewed to ensure accuracy of the diagnosis. In some of these cases, slides were reviewed by more than one pathologist.

**Table 1:** Summary of 201 camelid neoplastic and proliferative lesions diagnosed at CSU-VDL (1995–2020).

**Neoplastic/proliferative lesion**	**Number/species**	**Sex**	**Age**	**Location(s)**	**Percent of total cases**
EPITHELIAL	**77**				**38.3%**
Benign	**22**				**10.9**
** Cutaneous**					
**Fibropapilloma**	3 alpaca 1 llama	2 F 2 M	10 m, 8 y, 13 y, U	Hip; fetlock; tip of nose; gum and lip	**2.0**
Pilomatricoma^*^	1 alpaca 1 cam	2 M	10 yr; U	Lumbar skin	**1.0**
** Glandular**					
Pituitary adenoma^*^	2 alpaca 2 llama	1 F 3 M	11–20 y	Cortical adenohypophysis at pars distalis; chromophobe cystadenoma	**2.0**
**Thyroid adenoma**	3 llama	3 M	20 y, 21 y, 23 y	Thyroid; one cystadenoma	**1.5**
**Biliary cystadenoma**	1 alpaca 2 llama	1 F 2 M	6–21 y	Liver	**1.5**
**Mammary adenoma**	2 alpaca	1 M 1 U	1 week; U	Mammary gland	**1.0**
**Adrenal cortical adenoma**	1 alpaca	F	3 y	Adrenal gland cortex	**0.5**
Pancreatic cystadenoma^*^	1 llama	F	20 y	Pancreatic ducts, multilocular	**0.5**
Salivary gland cystadenoma^*^	1 alpaca	F	Un	Cervical region, biopsy	**0.5**
**Keratinizing ameloblastoma**	1 alpaca	M	6 y	Maxilla, upper dental arcade	**0.5**
Malignant	**55**				**27.4**
Squamous cell carcinoma	**19**				**9.5**
**Cutaneous**	4 alpaca 4 llama 1 cam	7 F 2 U	2–20 y	Sternum; trunk; perineum; limbs; dorsum	
**Ocular**	1 llama	M	15 y	Enucleated eye remnant, right eye	
**Oral**	3 alpaca	2 F 1M	7–11 y	Gingival mucosa with invasion into alveolar bone and lymphatics	
**Visceral**	2 alpaca 1 llama	1 F 2 M	2.2 y, 18 y, 25 y	Stomach; small bowel; esophagus	
**Unknown**	3 llama	2 M 1 U	10 y		
Intestinal adenocarcinoma	6 alpaca 4 llama	6 F 3 M 1 U	6–23 y	Small and large intestine; one case of carcinomatosis	**5.0**
Mammary adenocarcinoma	3 alpaca 4 llama 1 cam	7 F	7–18 y; U	Mammary gland w/metastasis to; lung, kidney, liver; lungs, rectum, adrenal gland, lymph nodes, liver; one case of carcinomatosis	**4.0**
Uterine adenocarcinoma	3 alpaca 1 llama	4 F	1–23 y	Uterus with metastasis to, lung, lymph nodes; one case of carcinomatosis	**2.0**
Renal carcinoma	2 alpaca 2 llama	2 F 2 M	15–22 y	Kidney	**2.0**
Pulmonary carcinoma	2 alpaca	1 F 1 M	14 m-14 y	Lung	**1.0**
Undifferentiated carcinoma	2 llama	1 F 1 M	18–19 y	Perineal skin; C3	**1.0**
Thyroid carcinoma	1 alpaca 1 llama	2 M	4 y; 23 y	Thyroid	**1.0**
Thymoma	1 llama	M	2.2 y	Thymus	**0.5**
Malignant pheochromocytoma^*^	1 llama	M	24 y	Adrenal gland medulla	**0.5**
Hepatic carcinoma	1 llama	M	4 y	Liver	**0.5**
Sebaceous carcinoma	1 cam	F	5 y	Neck	**0.5**
MESENCHYMAL	**53**				**26.5**
**Benign**	**33**				**16.4**
Fibroma	9 alpaca 5 llama 2 cam	10 F 4 M 2 U	3–6 y (A) 7–16 y (L)	Oral; nostrils; lip; limbs Oral; nostrils; limbs; eyelid; skin/hoof interface	**8.0**
Hemangioma	5 alpaca 1 llama	5 F 1 M	9–23 y	Lung; liver; mesentery; pericardium; C2; dermis; eyelid	**3.0**
Lipoma	2 alpaca 2 llama	1 F 3 M	11 y, 8 y, 23 y, 12 y	Near the prepuce, liver, C3	**2.0**
Leiomyoma	1 alpaca 2 llama 1 cam	3 F 1 M	6–20 y, U	Greater curvature of C3; uterus; rectum	**2.0**
Sarcoid	2 alpaca	2 F	4 y, U	lip	**1.0**
Myxoma^*^	1 llama	M	8 y	Medial canthus of the right eye	**0.5**
**Malignant**	**20**				**10.0**
Soft tissue sarcoma	4 alpaca 4 llama	6 F 2 M	6 wk −15 y (A) 1 y−20 y (L)	Lip including sarcoid-like tumor; nose; urethra	**4.0**
Sarcoma	3 alpaca 1 llama	4 F	7–20 y	Trachea, middle third; respiratory system and thoracic LN, pancreas; C3; elbow; lip commissure	**2.0**
Hemangiosarcoma	2 alpaca 1 llama	3 F	2 y 22 y, 23 y	Eyelid subcutis Mesentery; spleen and liver	**1.5**
Osteosarcoma^*^	2 llama	M F	6 y 19 y	Maxillary tooth root left jaw; humerus	**1.0**
Myxosarcoma^*^	1 llama	F	U	Right nare, mucocutaneous junction	**0.5**
Liposarcoma (grade 3)^*^	1 alpaca	F	6 y	Ventral chest subcutis	**0.5**
Theca cell tumor	1 alpaca	F	23 y	ovary	**0.5**
ROUND CELL NEOPLASIA	**43**				**21.3**
Lymphoma	27 alpaca 7 llama 2 cam	21 F 11 M 3 U	>1–20+ years	Lymph nodes, liver, lung, spleen, pharynx, C1, C3, kidney, spiral colon, uterus, urinary bladder, meninges, skeletal muscle, duodenum, mediastinum, bone marrow, mammary gland, myocardium, brain, eyelid, ovary, pancreas, thoracic duct, heart, peri-hilar lymph nodes, pleura	**18.0**
Cutaneous mast cell tumor	3 llama 1 cam	3 F 1 U	4 y, 10 y, 12 y	Labial subcutis; unspecified	**2.0**
Leukemia	2 alpaca	2	3 y, geriatric	Liver, spleen; bone marrow, kidneys, lymph nodes, lung, liver, spleen	**1.0**
Histiocytic sarcoma^*^	1 alpaca	F	4 y	Intrapelvic mass diagnosed at 2 weeks of age disseminated into mediastinum	**0.5**
Myeloproliferative disorder	1 alpaca	F	8 y	Spleen and liver	**0.5**
NEURAL TUMORS	**1**				**0.5**
Gemistocytic astrocytoma	1 alpaca	F	4 y	Left cerebral hemisphere	**0.5**
NEUROECTODERMAL	**10**				**4.9**
Epithelial					
**Neuroendocrine carcinoma**	2 alpaca 2 llama	1 F 3 M 1 F	7–13 y (L) 2–22 y (A)	Hepatic; multicentric; paravertebral; periaortic lymph nodes; spleen, mesenteric lymph nodes	**2.0**
Non-epithelial					
**Melanoma**	2 alpaca 3 llama	2 F 3 M 2 F	2 y, 13 y, U	Perineal skin; ocular; fetlock; lymph nodes; thyroid gland; lungs	**2.5**
**Congenital melanocytic nevus with malignant transformation**	1 alpaca	U	1 m	Periarticular skin	**0.5**
PROLIFERATIVE (NON-NEOPLASTIC) LESIONS	**16**				**8.5**
Adrenal hyperplasia	5 alpaca 3 llama 1 cam	6 F 2 M 6 F 1 U	4–23 y U	Adrenal gland cortex	**4.5**
Hamartoma	2 alpaca	2 F	4–5 y	Skin; smooth muscle in esophagus	**1.0**
Pituitary hyperplasia^*^	1 alpaca	M	6 y	Pituitary gland with leukoencephalopathy	**0.5**
Urinary bladder polyp^*^	1 alpaca	F	8 y	Urinary bladder mucosa	**0.5**
Lobular hyperplasia	1 alpaca	F	12 y	Mammary gland	**0.5**
Hybrid follicular cyst	1 alpaca	M	U	Lip, multiple	**0.5**
Cystic endometrial hyperplasia	1 llama	F	21 y	Uterus	**0.5**

* *Previously unreported to occur in camelids.*

*A, alpaca; L, llama; Cam, camelids; F, female; M, male; U, unknown; w, weeks; m, months; y, years; NA, not applicable.*

*The bolded terms are the summary case numbers. They equal all the cases below them added together. For example: Epithelial 77 = summary from benign through sebaceous carcinoma. It is the total number of epithelial neoplasia*.

### Statistical Analysis

To assess if a specific diagnosis occurred more frequently in llamas vs. alpacas for CSU-VDL cases, a two-sided Fisher's exact *t*-test was conducted to compare categories of neoplastic lesions. A *p* < 0.05 was considered significant. If the *t*-test was significant, an odds ratio was calculated to compare the likelihood of neoplastic/proliferative lesions in alpacas compared to llamas.

### Literature Review

For identification of literature reporting neoplasia in new world camelids, database search was performed, and 86 reports published between 1974 and 2020 were identified. These reports described proliferative and/or neoplastic lesion/s in llamas, alpacas, and/or guanaco. These reports are summarized in Table 2.

**Table 2:** Llama and alpaca neoplastic and proliferative lesion literature review 1974–2020.

**Neoplastic/proliferative lesion**	**Number/species**	**Location(s)**	**References**	**Year**
EPITHELIAL				
**Benign**				
Follicular				
**Follicular cysts (hybrid)**	4 alpaca	Dorsum; lateral neck; thorax; H. legs	(4)	2010
**Hybrid follicular cysts**	3 alpaca	Widespread especially trunk	(5)	2011
**Trichopeithelioma**	1 alpaca	Cranial thorax; dorsum; ventral neck	(6)	2005
**Trichopeithelioma**	1 alpaca	Neck; thorax; rump	(5)	2011
Fibropapilloma	3 alpaca 2 llama	Lip; cheek Lip; nares; nose	(7)	2003
Papilloma/fibropapilloma	2 alpaca	Lips; pasterns	(5)	2011
Transitional cell papilloma	1 alpaca	Renal pelvis	(8)	2008
Corneal epithelial inclusion	1 llama	Right eye limbus	(9)	2008
Pituitary adenoma	1 alpaca	Pituitary gland	(10)	2012
Pituitary null cell adenoma	1 llama	Pituitary gland	(11)	2014
Hepatocellular adenoma	1 llama	Liver	(12)	1994
**Malignant**				
Squamous cell carcinoma				
**Cutaneous**	1 llama	Sternum	^**^(13)	1989
**Cutaneous**	1 llama	Cutaneous scar on thorax to paralumbar fossa	(14)	1997
**Cutaneous**	4 llama	Perineum; trunk; limbs	(3)	2007
**Cutaneous**	1 alpaca	Sternal callosity with axillary lymph node and lung metastasis	(15)	2020
**Ocular**	1 alpaca 1 llama	Third eyelid	(3)	2007
**Mammary**	1 llama	Cutaneous metastasis	(16)	2001
**Mammary**	2 llama	Metastatic to lymph nodes, disseminated	(3)	2007
**Gastric**	1 guanaco	C1	^*^(1)	1974
**Gastric**	1 llama	C1	(17)	1988
**Gastric**	1 llama	C1	^**^(13)	1989
**Gastric**	3 llama	C-3; C-1; junction of C1–C2	(18)	1997
**Gastric**	1 llama	Gastric	(19)	2012
**Gastric**	1 alpaca	C1 with carcinomatosis	(20)	2020
Adenocarcinoma				
**Intestinal**	1 llama	Disseminated	(3)	2007
**Intestinal**	1 alpaca	Intestinal with carcinomatosis	(21)	2020
**Uterine**	1 llama	Widespread metastasis	^**^(13)	1989
**Uterine**	1 llama	Uterus	(22)	1990
**Uterine**	1 llama	Widespread metastasis	(23)	2009
**Biliary**	2 llama	Disseminated	(3)	2007
**Biliary**	1 llama	Liver with widespread metastasis	(24)	2012
**Biliary**	1 alpaca	Liver with lung metastasis	(2)	2019
**Pancreatic**	1 alpaca	Disseminated	(3)	2007
**Mammary**	1 llama	Metastasis to sublumbar lymph nodes	(13)	1989
**Mammary**	1 llama	Carcinoma in a mixed mammary tumor	(25)	2007
**Mammary**	1 alpaca 1 llama	Disseminated	^**^(26)	2006
**Pulmonary**	1 llama	Metastatic disseminated	(27)	2002
**Pulmonary**	2 llama	One disseminated, one intrapulmonary	(28)	2004
**Pulmonary**	1 alpaca	Multifocal all lung lobes	(29)	2019
**Pulmonary**	1 alpaca	Not specified	(15)	2020
**Thyroid**	1 llama	Bilateral thyroid glands with lymph node metastasis	(30)	2019
Transitional cell carcinoma	1 llama	Renal papillae	(31)	1995
Granulosa-theca cell tumor	1 llama	Right ovary	(32)	2010
Granulosa cell tumor	1 alpaca	Ovary	(33)	2005
Anaplastic carcinoma	1 alpaca	Suspect adrenal cortical	(33)	2005
Un-differentiated carcinoma	1 alpaca	Disseminated	(3)	2007
MESENCHYMAL				
**Benign**				
Lipoma	2 llama	Mesentery; subcutis	(3)	2007
Fibroma	2 alpaca	Lower lip	(5)	2011
Ossifying fibroma	1 llama	Skull rostral and ventral to left canthus	(34)	2000
**Malignant**				
Fibrosarcoma	1 alpaca 3 llama	Lip; gingiva; maxilla; cornea	(3)	2007
Fibrosarcoma	1 alpaca	Lip	(5)	2011
Fibrosarcoma	1 alpaca	Maxillary with osseous metaplasia	(35)	2015
Stromal cell sarcoma	1 llama	Mandible with osteolysis	(33)	2005
Leiomyosarcoma	1 llama	Uterus	(3)	2007
Leiomyosarcoma	1 alpaca	Pelvic canal, urogenital	(36)	2010
Sarcoma	1 alpaca	Urethra	(37)	2005
Rhabdomyosarcoma	1 alpaca	Shoulder with lymph node metastasis	^**^(38)	2013
Hemangiosarcoma	1 llama	Liver	(12)	1994
Intraosseous hemangiosarcoma	1 llama	Left front leg (distal end)	(39)	1997
Anaplastic sarcoma	1 llama	Mandible	(40)	1996
Interstitial cell tumor	1 alpaca	Ovary—hyperandrogenism	(41)	2006
Interstitial cell tumor	1 alpaca	Ovary	(3)	2007
Sertoli cell tumor	1 alpaca	Right testicle	(42)	2020
ROUND CELL NEOPLASIA				
Lymphoma				
**Subcutaneous**	1 alpaca	Widespread	(5)	2011
**Epitheliotropic cutaneous T-cell**	1 alpaca	Cutaneous; perioral, periocular, and periauricular tissue, axillae, ventral abdomen	(43)	2016
**Visceral lymphoma and primitive malignant round cell tumors (PMRCT)**	1 llama	Disseminated	(44)	1985
“ ”	2 llama	Disseminated	^**^(13)	1989
“ ”	1 llama	Multicentric	(45)	1993
“ ”	1 llama	Liver and kidney	(12)	1994
“ ”	7 llama 3 alpaca	Lymph nodes; liver; kidneys; lungs	^**^(46)	1995
“ ”	1 alpaca	Liver and kidneys	(47)	2001
“ ”	2 alpaca	Disseminated	(48)	2002
“ ”	3 alpaca	Multicentric	(49)	2004
“ ”	2 alpaca 1 llama	Not mentioned “ ”	(33)	2005
“ ”	1 alpaca	“ ”	(50)	2006
“ ”	4 alpaca 1 llama	Multicentric Multicentric	(3)	2007
“ ”	5 alpaca 1 llama	Disseminated; lymph nodes; liver; kidneys	(51)	2008
“ ”	12 alpaca 12 llama	Multicentric; gastric; abdominal; thoracic; focal Multicentric; gastric; abdominal; thoracic	(52, 53)	2009 2010
“ ”	1 alpaca	Disseminated	(54)	2010
“ ”	1 alpaca	Disseminated—bovine leukemia virus positive	(55)	2012
“ ”	20 alpaca 6 llamas	Disseminated; lymph nodes; liver; kidneys; abdominal mass; spleen; mammary; gastrointestinal; lung; heart	^**^(56)	2013
“ ”	1 alpaca	Disseminated—cerebral involvement	(57)	2013
“ ”	1 alpaca	Abdominal mass	(58)	2015
“ ”	4 alpaca	Not mentioned	(2)	2019
“ ”	1 alpaca	Tracheal	(59)	2020
**Acute myeloid leukemia**	1 alpaca	Disseminated	(60)	2008
**Cutaneous mast cell tumor**	1 llama	Right hip; left cheek; right and left shoulders	(61)	2010
NEURAL TUMORS				
Gemistocytic astrocytoma	1 llama	Medullary with vestibular disease	(62)	1990
NEUROECTODERMAL				
Epithelial				
**Neuroendocrine tumor**	1 alpaca	Disseminated (liver, kidney, lymph nodes)	(49)	2004
Non-epithelial				
**Cutaneous melanocytoma**	1 llama	Left axilla	(63)	2005
**Cutaneous melanocytoma**	1 llama	Pectoral skin	(3)	2007
**Cutaneous melanocytoma**	1 alpaca	Right lower eye lid	(5)	2011
**Osteogenic melanoma**	1 alpaca	Right eye	(64)	2009
**Malignant melanoma**	1 alpaca	Left submandibular (cervical)	(65)	2009
TERATOID AND PRIMITIVE TUMORS				
Intracranial teratoma	1 alpaca	Temporal lobe	(66)	2008
Renal teratoma	1 llama	Right kidney	(67)	2004
Teratoid medulloepithelioma	1 llama	Enucleated eye	(68)	2000
Medulloepithelioma	1 llama	Eye	(69)	1997
Non-teratoid medulloepithelioma	1 llama	Right eye	(70)	2006
Retinoblastoma	1 llama	Left eye	(71)	2005
Ameloblastoma	1 llama	Mandible	(72)	2006
Acanthomatous ameloblastoma	1 alpaca	Right maxilla	(73)	2005
**Keratinizing ameloblastoma**	1 llama	Right caudal face	(74)	2004
**Ameloblastic odontoma**	1 llama	Rostral mandible	(75)	2003
Hepatoblastoma (congenital)	1 alpaca	Liver right lateral lobe	(76)	2001
Nephroblastoma	1 guanaco	Right kidney; lungs; liver	(77)	1988
Primitive stromal tumor	1 alpaca	Testicle	(3)	2007
Trophoblastic tumor	1 llama	Uterus	^**^(22)	1990
PROLIFERATIVE (NON-NEOPLASTIC) LESIONS				
Thyroid nodular hyperplasia	1 llama	Thyroid glands	(78)	2003
Collagen and hair follicle hamartoma	1 alpaca	Eyelid; neck; foot	(5)	2011
Aneurysmal bone cysts	1 llama	Multifocal polyostotic	(79)	1997
Periradicular cyst	1 alpaca	Right mandible	(80)	2011
Polycystic liver	2 alpaca	Liver	(81)	2013
Polycystic liver	2 llama	Liver	(82)	2019
Cystic rete testes	1 alpaca	Bilateral testicles	(83)	2006
Bladder polyp	1 alpaca	Trigone of the bladder	(15)	2020

*C1, C2, and C3 = gastric chambers 1, 2, and 3.*

** *Previously published cases from the CSU Veterinary Diagnostic Laboratory.*

* *First published report of neoplasia in a new world camelid*.

## Results

### Neoplasia Overview

#### CSU-VDL Cases

**Figure 1:**
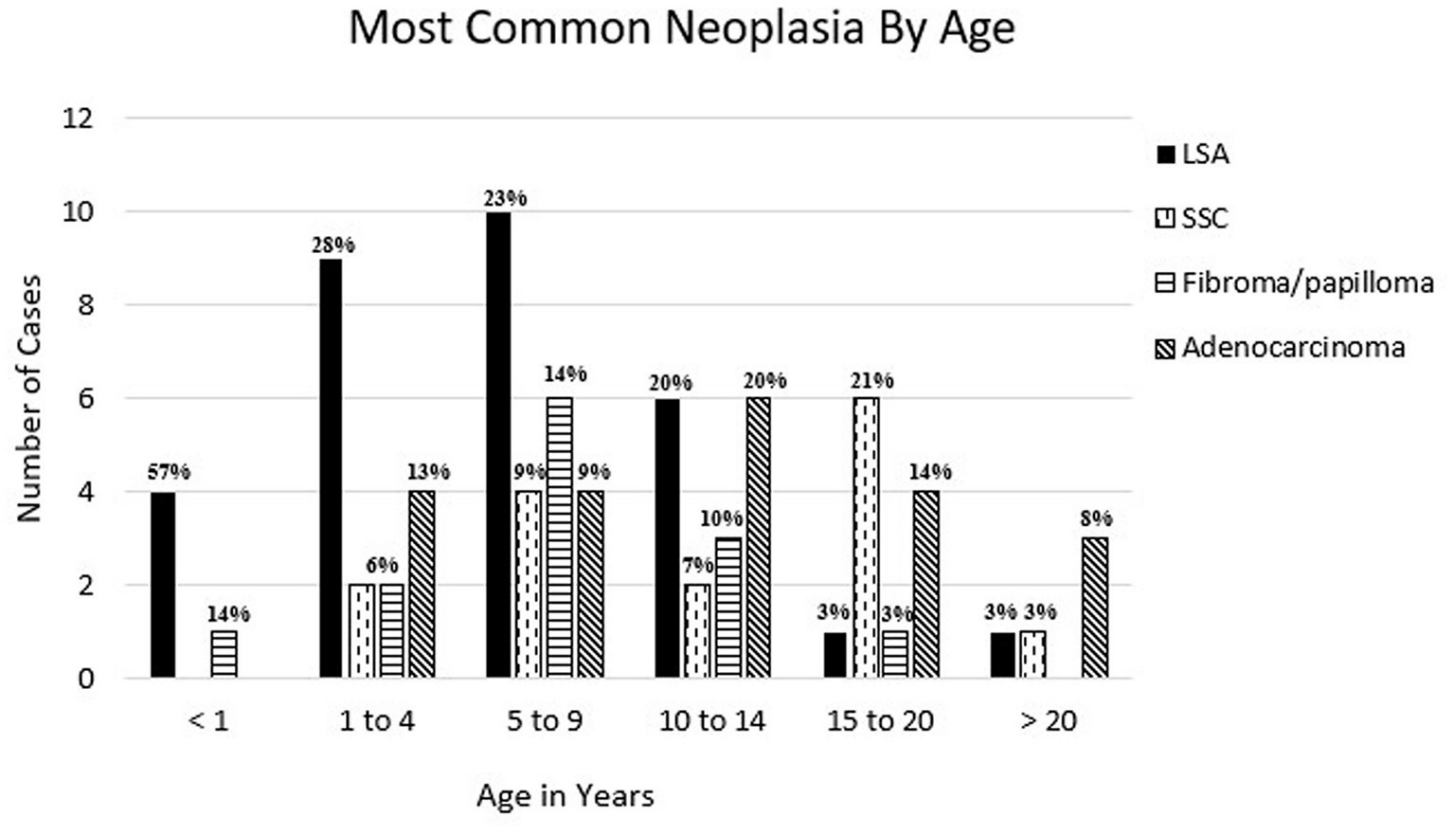
Most common reported neoplasia in llamas and alpacas by age at CSU-VDL from 1995 to 2020. Percentage of total cases per age is reported above bar. LSA, lymphoma; SCC, squamous cell carcinoma; adenocarcinoma = mammary, gastrointestinal, uterine, pulmonary, thyroid, and unspecified.

Two hundred and one diagnoses of proliferative lesions or neoplasia from 192 animals were identified from 2,166 total submissions to the CSU-VDL from 1995 to 2020 representing 8.8% of all camelid cases submitted for biopsy or necropsy. Sixteen cases were classified as proliferative (<1% of all submitted cases) and 185 cases diagnosed as benign or malignant neoplasia (8.5% of all submitted cases). Cases are summarized in Table 1. Of these cases, 57% were from alpacas and 36% were from llamas with the remaining cases only identified as camelid. Of the proliferative or neoplastic lesions, 59% occurred in females, 34% in males, and the remaining cases have an unreported sex. The percentage of lesions in animals <1 year in age was 3.5%, 1–4 years of age was 16%, 5–9 years of age was 21%, 10–14 years old was 14.5%, and >15 years of age was 25% (age was unknown in 18% of cases). The most common tumors diagnosed per age range are shown in Figure 1. Mesenchymal tumors were found to be more likely than epithelial tumors to be malignant (*p* = 0.0174).

#### Literature Cases

Two-hundred and eighteen proliferative or neoplastic lesions have been reported in the literature since the first report of squamous cell carcinoma in a guanaco in 1974 (1). Of these cases, 54% are reported in alpacas, 45% in llamas, and 1% in guanaco. There has been one previous summary report of causes of mortality in 100 alpacas which reported 5% rate of neoplasia (four cases of lymphoma and one case of cholangiocellular carcinoma) (2). There is one review of 40 cases of neoplasia in alpacas and llamas that found a rate of 4.9% of neoplasia in alpacas and 11% of neoplasia in llamas (3).

### Round Cell Neoplasia

#### Lymphoma

##### CSU-VDL Cases

From 1995 to 2020 CSU-VDL, a total of 36 cases were diagnosed as lymphoma or lymphoproliferative disease, more than any other single type of neoplasia. These cases represented 18% of all neoplastic and proliferative diseases diagnosed. Alpacas were significantly overrepresented with 27 cases vs. 7 in llamas (2 cases only identified as camelid) (*p* = 0.0192). The odds ratio of lymphoma compared to all other neoplastic/proliferative lesions in alpacas vs. llamas is 2.904 (95% CI: 1.213–7.555). Lymphoma was overrepresented in 21 (75%) females vs. 11 (31%) males with 3 cases being of unreported sex. Additionally, lymphoma was also the most common neoplasia to be diagnosed in animals under the age of 10 with it representing 57, 28, and 23% of diagnoses in animals <1, 1–4, and 5–9, respectively (Figure 1).

##### Literature Cases

Literature review identified 109 cases of lymphoma reported in llamas and alpacas with most of these cases occurring in alpacas (74/109; 68%). The report by Valentine et al. reports only 12.5% of all neoplastic cases being lymphoma (5 out of 40 cases), and it was the third most common tumor following fibromas/fibropapillomas/fibrosarcomas and squamous cell carcinomas (3). In the published cases, lymphoma generally presented widely disseminated with rare reports of cutaneous and solitary masses (5, 43, 59). Phenotyping by immunohistochemistry in 63 of these cases identified 24 B cell (38%), 28 T cell (44%), 1 mixed T and B cell, and 13 (20.6%) as non-B, non-T cell lymphoma (43, 48–52, 54–59).

#### Other

##### CSU-VDL Cases

Other round cell tumors were diagnosed much less frequently in CSU-VDL submissions. There were four mast cell tumors identified through the CSU-VDL submissions representing 2.0% of the total proliferative and neoplastic lesions. Metastasis was not found for any of these tumors. Additionally, there was a single diagnosis of disseminated histiocytic sarcoma (previously unreported in the literature).

##### Literature Cases

In the literature, there is only one report of multiple mast cell tumors in a llama and metastasis was not reported (61).

### Epithelial Neoplasia

#### CSU-VDL Case Summary

Epithelial neoplasia represented 38.3% of cases with an average age of 13.4 years. There was approximately the same number of epithelial neoplasia diagnosed in llamas vs. alpacas (35 vs. 38 cases, respectively), and females were slightly overrepresented with 48% of all diagnoses vs. 42% males (in the remainder of cases, the sex was not reported).

#### Follicular and Benign Tumors

##### CSU-VDL Cases

Follicular cysts and tumors were only rarely diagnosed from CSU-VDL submissions. These lesions include follicular cysts and pilomatricoma. Both cases occurred in alpacas. This is the first report of a pilomatricoma in a new world camelid.

**Figure 2:**
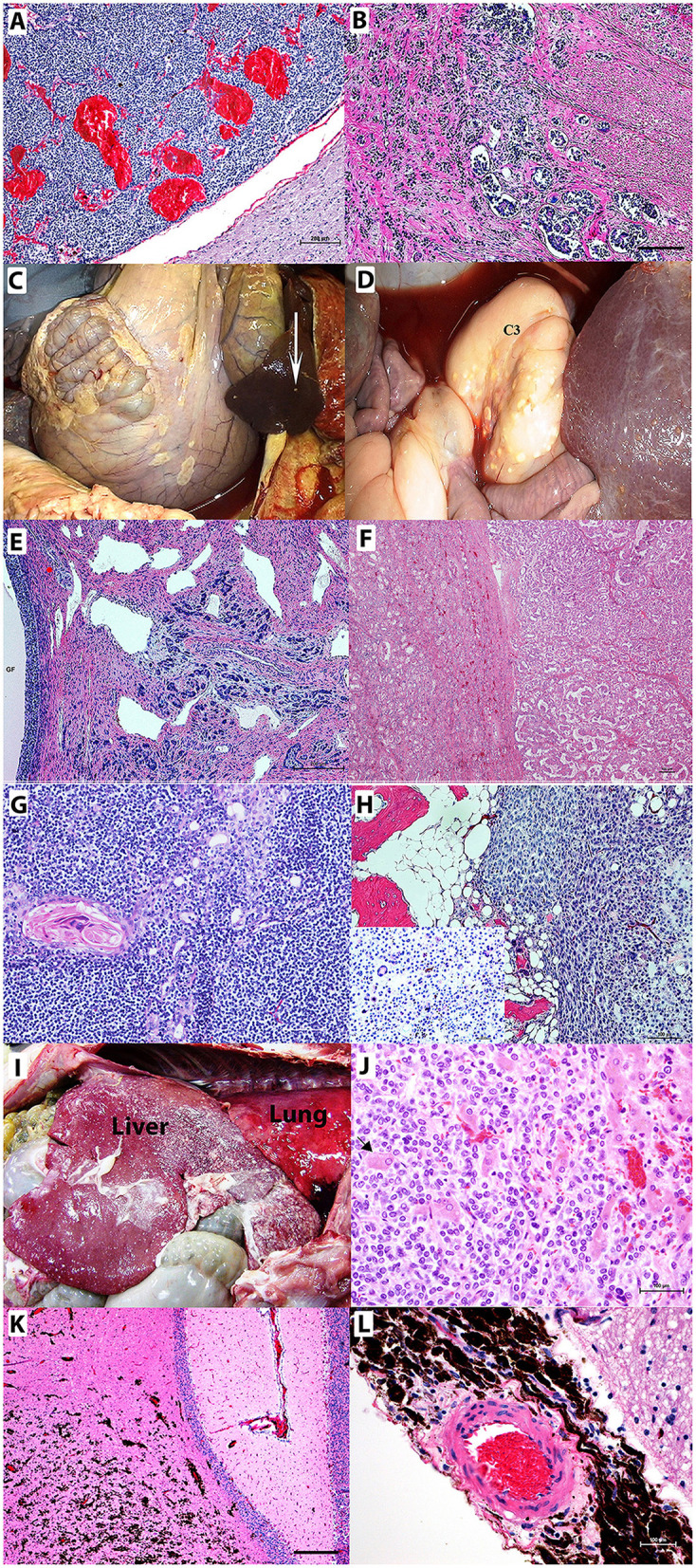
Neoplastic lesions in camelids. **(A)** Pituitary adenoma. Non-functional chromophobe pituitary adenoma replacing a large part of pars distalis composed of homogenous populations of small round basophilic cells in the midst of congested vascular sinuses. Bar = 200 μm **(B)** C3 adenocarcinoma with epithelial cells exhibiting invasive growth into the submucosa and muscularis with lymphatic tumor emboli. Bar = 200 μm **(C,D)** Abdominal carcinomatosis from C3 adenocarcinoma involving mesentery, intestines, spleen (arrow), liver, and third gastric chamber (C3) appears as plaques or nodules on surfaces of involved organs. **(E)** Uterine adenocarcinoma with carcinomatosis spreading to the ovary containing large graafian follicle (GF) Bar = 200 μm. **(F)** Renal carcinoma exhibiting a tubular pattern. **(G)** Lymphocyte-rich thymoma in a llama showing concentric clusters of epithelial cells with abundant eosinophilic cytoplasm, Hassall's corpuscles, and predominantly surrounding small lymphocytes. **(H)** Humeral osteosarcoma and giant cell osteosarcoma. Neoplastic cells surround a scant amount of eosinophilic osteoid. Inlay shows multinucleated giant cells. **(I,J)** Neuroendocrine carcinoma with invasive growth throughout the diaphragmatic surface of the liver. The liver also has multifocal accumulations of fibrin. **(I)** Multifocal hepatocytes are entrapped [**(J)** arrow] by the neoplastic cells. **(K,L)** Infiltrating the cerebellar neurophil **(K)** and surrounding the meningeal artery **(L)** are metastatic melanocytes in a newborn alpaca with a congenital nevus that has undergone malignant transformation. Hematoxylin and eosin staining. Bars = 100 μm unless otherwise noted.

There were several proliferative lesions and cases of benign epithelial neoplasia diagnosed. The most common is adrenal cortical hyperplasia (nine cases) followed by fibropapillomas/papillomas (four cases) and pituitary adenomas (four cases) (Figure 2A) with one case of pituitary hyperplasia.

##### Literature Cases

Follicular cysts and tumors are reported more commonly in the literature than were diagnosed in the CSU-VDL submissions and occurred more often in alpacas (nine cases) than llamas (zero cases) (4–6). None of the follicular lesions showed any indication of malignancy or aggressive behavior.

There are two reports of pituitary adenomas with one case causing brain compression and neurologic signs (10, 11). Fibropapillomas have been identified as one of the most commonly reported lesions in new world camelids (3). (In one report, these tumors were combined with fibroma, making determination of exact numbers of fibropapillomas difficult to determine.) These lesions generally presented on the face with occasional development on the distal limbs.

#### Squamous Cell Carcinoma

##### CSU-VDL Cases

There were 19 (9.5% of all neoplastic and proliferative lesions) diagnoses of squamous cell carcinoma from CSU-VDL submissions, making them the second most common neoplasia behind lymphoma. The most common locations were cutaneous (nine cases) and oral (three cases) with single cases reported in the eye, esophagus, gastric compartment, and small intestine. Only two cases were associated with metastasis (22.2%).

##### Literature Cases

Squamous cell carcinoma was the first reported neoplasia in new world camelids, and it has been reported as one of the most common tumors to affect these animals (1, 3). The most common sites of occurrence were the gastric chamber one followed by skin, mammary gland, and third eyelid. Approximately a third of the squamous cell carcinomas were reported to have metastasized to regional lymph nodes and/or distant sites with one report of extensive carcinomatosis within the abdominal cavity (20).

#### Adenocarcinoma

##### CSU-VDL Cases

Adenocarcinomas were a commonly diagnosed neoplasia in both llamas and alpacas in the CSU-VDL submissions (22 cases, 11% of all neoplastic or proliferative lesions). The majority of these cases were associated with the gastrointestinal tract (10/22 cases, 45.5%), and all had metastasis at the time of diagnosis to the local lymph nodes, liver, spleen, and/or the lungs. There was one case of carcinomatosis associated with a gastrointestinal adenocarcinoma (Figures 2B–D). Female camelids were more likely to be affected by adenocarcinomas due to a high number of mammary (seven cases, 31.8%) and uterine tumors (four cases, 18.2%). Two of the six mammary carcinomas were found to have metastasized at the time of diagnosis along with two of the three uterine adenocarcinomas including one case of carcinomatosis to the ovaries and peritoneal cavity (Figure 2E).

##### Literature Cases

In the literature, adenocarcinomas were associated with an aggressive disease outcome as 18/20 adenocarcinomas had established disseminated or metastatic disease at the time of diagnosis. Additionally, there was one case of gastrointestinal adenocarcinoma associated with carcinomatosis reported in the literature (21).

#### Other

##### CSU-VDL Cases

There were epithelial tumors diagnosed at the CSU-VDL that have never or very rarely before been reported in camelids. These include renal carcinomas (four cases), thyroid carcinoma (2 cases), and thymoma (one case). Renal carcinomas occurred in older animals (average age 19), and all were tubular in pattern (Figure 2F). The thymoma was lymphocyte rich and occurred in a 2-year-old male llama (Figure 2G).

### Mesenchymal Neoplasia

#### CSU-VDL Case Summary

Mesenchymal neoplasia represented 27% of cases with an average age of 12.2 years. Alpacas and females were overrepresented with 57 and 72% of mesenchymal neoplasia, respectively.

#### Benign

##### CSU-VDL Cases

From the CSU-VDL submissions, fibromas were the third most diagnosed tumor in camelids (16 cases, 8%). There were also many hemangiomas (6 cases). Hemangiomas tended to have visceral (liver, pericardium, lung) involvement and were diagnosed in older animals (average age 18 years) vs. fibromas which were cutaneous and generally occurred in younger animals (average age 8.5 years). Other reported benign mesenchymal tumors include leiomyoma (stomach chamber 3 [C3], uterus, and rectum), lipoma (subcutaneous, C3 causing obstruction, liver, and prepuce), and two cases of sarcoid. Additionally, there was one Sertoli cell tumor diagnosed at CSU-VDL in an alpaca.

##### Literature Cases

Valentine et al. reported fibromas/fibropapillomas as the most diagnosed lesion in new world camelids (3). Other benign mesenchymal tumors reported in the literature include two reports of ovarian interstitial cell tumors and one report of a Sertoli cell tumor in an alpaca (3, 41, 42).

#### Malignant

##### CSU-DVL Cases

Of the 53 mesenchymal tumors identified from CSU-VDL submissions, 19 were diagnosed as sarcomas (17% of mesenchymal tumors and 9.5% of all proliferative and neoplastic lesions). The average age of diagnosis was 10.3 years (range 6 weeks−23 years), 84% (16 of 19 cases) occurred in female animals, and sarcomas were almost evenly distributed between llamas and alpacas (9 cases vs. 12 cases). The most common diagnosed sarcomas were fibrosarcomas or soft tissue sarcomas (8/19) that typically occurred in the oral cavity. They were also reported to occur in the nose, trachea, subcutaneous adipose tissue, C3, and arising from the pancreas (reported to have metastasized to the local lymph nodes). There was one unusual case of a sarcoma arising from the urethra of a 6-week-old female alpaca. There were three diagnoses of hemangiosarcoma, two visceral (liver and spleen) and one cutaneous (eyelid), and two cases of osteosarcoma both arising from the jaw (one from a tooth root abscess and the other from a giant tumor of bone) (Figure 2H). Splenic, disseminated, and visceral hemangiosarcomas are herein reported for the first time.

##### Literature Cases

Fibrosarcomas are commonly reported in the literature and similar to the CSU-VDL cases most commonly reported in the oral cavity (3, 5, 33, 35). Two cases of hemangiosarcoma have been reported, one arising from the liver and one intraosseous hemangiosarcoma (12, 39).

#### Other

##### CSU-VDL Cases

There were four cases of neuroendocrine carcinomas and five melanocytic tumors identified through the CSU-VDL submissions representing 2.0 and 2.5% of total proliferative and neoplastic lesions, respectively. All neuroendocrine carcinomas were highly aggressive with metastasis at the time of diagnosis, making definitive identification of the origin difficult to determine (Figures 2I,J). Four of the five melanocytic tumors had metastases at the time of diagnosis with the most common sites of metastases being local lymph nodes and the lungs. There was a single case of a congenital melanocytic nevus with malignant transformation and metastasis to the brain and lungs in a 1-month-old alpaca, suggesting that it was congenital (Figures 2K,L).

Primitive and neuronal tumors were rare. There was one case of keratinizing ameloblastoma diagnosed and one case of gemistocytic astrocytoma diagnosed in a 4-year-old female alpaca in the CSU-VDL submissions.

##### Literature Cases

Neuroectodermal neoplasia are rarely reported with one case of neuroendocrine carcinoma (49) and five reports of melanoma (3, 5, 63–65). Melanomas have been reported to metastasize.

Primitive and neural tumors are rarely reported. Primitive tumors include teratomas (cerebellum and kidney), medulloepitheliomas and retinoblastoma of the eye, ameloblastoma of the mandible, hepatoblastoma, nephroblastoma, primitive stromal tumors of the testicle, and trophoblastic tumor of the uterus (3, 22, 66–74, 76, 77). The only primary neural neoplasm reported was a gemistocytic astrocytoma in a llama (62).

### Other Non-neoplastic Proliferative Lesions

In addition to the benign and proliferative lesions previously discussed, there were a number of non-neoplastic proliferative lesions diagnosed in the CSU-VDL submissions and in the literature including polyps; hamartomas; bone cysts; polycystic liver; cystic rete testes; and thyroid, adrenocortical, lobular mammary, and endometrial hyperplasia (5, 15, 78–83).

## Discussion and Conclusions

Examination of archived camelid submission to CSU-VDL in the period between April 20, 1995 to December 31, 2020, confirms that neoplasia in camelids is heterogeneous and affects both alpacas and llamas of wide age ranges. The prevalence of neoplasia and proliferative lesions in both species is 8.8% of the total camelid pathology submissions to CSU-VDL, which is higher than the prevalence of camelid neoplasia reported in the literature from other regions (5%) (2, 3). The considerably longer duration of the study period and the higher number of total camelid submissions including more alpacas than llamas might be among the reasons for this variation.

In the South American camelid species, there was a female predisposition for developing neoplasia with 113 female alpacas and llamas (57% of the total cases) developing one or more neoplastic/proliferative lesions compared to 64 male camelids (32%). The relative commonality of mammary and uterine neoplasia and the relative scarcity of testicular tumors, in part, explain why female camelids might be overrepresented in the current review. The predisposition of female alpacas to develop non-genital neoplasia was also observed especially in the case of lymphoma (58% of lymphoma cases were diagnosed in females). Females also had a much higher prevalence of sarcomas (84%) than males in both alpacas and llamas.

Round cell tumors were by far the most commonly diagnosed/reported neoplasia in camelids with lymphoma being the top tumor diagnosed from the CSU-VDL. Interestingly, lymphoma was diagnosed much more commonly in alpacas (75%) than llamas (19%) (*p* = 0.0192) and the most common tumor diagnosed in animals <5 years of age (Figure 1). Lymphoma was most likely to be disseminated at the time of diagnosis and was the most common tumor to affect the brain. The high prevalence of lymphoma in alpacas and in younger animals suggests a possible genetic link, as is observed in humans and dogs (84). B vs. T cell lymphomas were evenly distributed in cases that had been further immunophenotyped. Also, there is a single case report of bovine leukemia virus-associated lymphoma in an alpaca (55). Evaluation of CSU-VDL cases for bovine leukemia virus was not performed but could be an interesting area for further investigation.

Other round cell tumors included mast cell tumor and histiocytic sarcoma. The CSU-VDL submissions have increased numbers of these tumors when compared to the literature. Histiocytic sarcoma is reported here for the first time in the alpaca species. It occurred in a very young cria, suggesting that the tumor might have developed *in utero* as it was of considerable size at 2 weeks of age. None of the mast cell tumors found in the CSU-VDL submissions were found to have metastasized.

Malignant epithelial tumors accounted for 69% of camelid epithelial neoplasia. Squamous cell carcinomas were the most common malignant epithelial neoplasia with an overall prevalence of 9.5%, and intestinal, mammary, and uterine adenocarcinomas had an overall prevalence of 27%. Locations of the squamous cell carcinomas included cutaneous, oral, ocular, and visceral. To the authors' knowledge, oral squamous cell carcinomas are a novel category; however, gastric squamous cell carcinoma, ocular-third eyelid squamous cell carcinoma, and squamous cell carcinoma arising from a cutaneous scar have previously been reported in the literature (1, 3, 14, 17, 18). Although the exact etiopathogenesis of the squamous cell carcinomas encountered in camelids is largely unknown, genetic make-up, environmental conditions, and infectious agents are among the possibilities (18). In cases from the CSU-VDL database, only two squamous cell carcinomas were found to have metastasized at the time of the diagnosis, indicating that complete excision of these tumors could be potentially curative in affected animals. Reports from the literature find these tumors to be more aggressive than our database suggests, but this may represent a skewed dataset where only novel and aggressive lesions are published (3, 15, 16, 20).

Adenocarcinomas have a much more aggressive clinical course with most cases having had metastasized at the time of diagnosis. Gastrointestinal tumors were the most common category with all having metastasized at the time of diagnosis and one case having developed widespread carcinomatosis identified in CSU-VDL cases and one reported in the literature (21). It is unknown if these tumors metastasize early or if the frequent metastasis is due to the difficulty in detecting these tumors early in the course of the disease. Diagnosis of adenocarcinoma also had an increased female prevalence. This is likely due to the diagnoses of uterine and mammary carcinomas. Uterine and mammary adenocarcinomas were found to have metastasized in 66 and 33% of cases, respectively, indicating increased incidence of metastasis when compared to squamous cell carcinomas but less than gastrointestinal adenocarcinomas. Other organs that were affected by adenocarcinomas/carcinomas include thyroid, liver (biliary carcinomas), and kidney.

Fibropapilloma is the most prevalent benign epithelial neoplasm. The prevalence of fibropapilloma diagnosed at CSU was higher than in the Cornell study but lower than in the Oregon study, as the authors of the latter study combined both fibropapillomas and fibromas into one category (3, 5). Interestingly, there is a single report of papillomavirus isolated from a llama fibropapilloma which was 73% homologous with bovine papillomavirus type 1 (BPV1) (7). Evaluation for BPV1 in the fibropapillomas diagnosed at CSU was not performed but is of interest for future investigation.

Mesenchymal neoplasia occurred in 52 camelids with an overall prevalence of 26.5, 36% of which were identified as malignant. Fibroma was the most common benign mesenchymal neoplasm at 30.8% of mesenchymal neoplasia and overall prevalence of 8%. There was an even distribution between llamas and alpacas diagnosed with sarcomas and a higher number of females than males. Fibrosarcoma or soft tissue sarcoma, especially of the oral or nasal cavity, was the most common diagnosis. Only two of eight were found to have metastasized at the time of diagnosis. In the literature, there are no reports of metastasis of these tumors, suggesting good prognosis following complete surgical excision. Other less common diagnoses included osteosarcoma, liposarcoma, myxosarcoma, and hemangiosarcoma. Only the visceral sarcomas (affecting the liver and spleen) were found to have metastasized similar to the aggressive behavior of these tumors in other species (85).

Several neoplastic and proliferative lesions, never reported, were identified from the CSU-VDL submissions. These include osteosarcoma, liposarcoma, myxosarcoma, cutaneous hemangiosarcoma, gastric leiomyoma, thymoma, pilomatricoma, sarcoid, malignant giant nevus, pituitary hyperplasia, adrenal cortical hyperplasia, and malignant pheochromocytoma. There were additionally many non-neoplastic and proliferative lesions identified. While these lesions are unlikely to be of clinical relevance, they are important differentials as they could mimic more aggressive neoplastic lesions using imaging modalities including ultrasound or radiographs, or even upon gross exam or necropsy.

The previously unreported congenital giant nevus with malignant transformation in a newborn alpaca is an interesting case. In this mass, there was abnormal cutaneous proliferation of melanoblasts present at birth in the periauricular skin and choristomas discoloring the lungs, liver, and meninges. A similar condition has been described in humans, in which giant congenital melanocytic nevi underwent malignant transformation (86, 87).

Here we summarize and review neoplastic/proliferative lesions in llamas and alpacas. Overall, the behavior of these tumors is similar to the behavior reported in other species, indicating that complete surgical excision may be curative in many incidences of proliferative and neoplastic lesions. There is a substantial risk of aggressive disease outcome and metastasis with certain types of neoplasia and with tumors in particular locations, indicating that staging of some patients is prudent. The current article will likely help to provide relevant prognostication to guide owners and clinicians of the possible outcome and best treatment plan. Risk factors appear to be female sex and increased age except in the case of lymphoma, which tends to occur in younger camelids. Lymphoma, melanomas, and adenocarcinomas (especially of gastrointestinal tract) carry an increased risk of dissemination in a single-organ system or widespread metastases. Sarcomas and squamous cell carcinomas in contrast are less likely to metastasize, and resection of these tumors may be curative in many cases. An important and interesting follow-up would be evaluation of disease-free interval and survival following medical intervention in camelids affected by the most common malignant tumors. In conclusion, camelid neoplasia constitutes a reasonable proportion of pathology submissions to veterinary teaching hospitals and veterinary diagnostic laboratories and, therefore, merits further studies to establish standards for their classification and prognostication.

## Data Availability Statement

The original contributions presented in the study are included in the article/supplementary material, further inquiries can be directed to the corresponding author/s.

## Author Contributions

AV: writing of manuscript, literature review, figures, and manuscript editing. MW: review and summary table Colorado State University cases. AH and JF: writing of manuscript and manuscript editing. TA: writing of manuscript, figures, and manuscript editing. All authors contributed to the article and approved the submitted version.

## Conflict of Interest

The authors declare that the research was conducted in the absence of any commercial or financial relationships that could be construed as a potential conflict of interest.

## Publisher's Note

All claims expressed in this article are solely those of the authors and do not necessarily represent those of their affiliated organizations, or those of the publisher, the editors and the reviewers. Any product that may be evaluated in this article, or claim that may be made by its manufacturer, is not guaranteed or endorsed by the publisher.
